# Rational Design and Immunological Mechanisms of Circular RNA-Based Vaccines: Emerging Frontiers in Combating Pathogen Infection

**DOI:** 10.3390/vaccines13060563

**Published:** 2025-05-26

**Authors:** Ying Zhang, Shumei Jin, Zan Zuo, Shujing Liu, Juan Xu, Chongyi Yang, Ping Wan, Linting Xun, Mei Luo, Fan Yang, Wenjie Chen, Zhengji Song, Jialong Qi

**Affiliations:** 1 Yunnan Provincial Key Laboratory of Clinical Virology, The First People’s Hospital of Yunnan Province, The Affiliated Hospital of Kunming University of Science and Technology, Kunming 650032, China; 20222236001@stu.kust.edu.cn (Y.Z.); zuozan007@163.com (Z.Z.); 2 School of Medicine, Kunming University of Science and Technology, Kunming 650504, China; xuiuan1@stu.kust.edu.cn (J.X.); kmwp_66@126.com (P.W.); yangfan@kmust.edu.cn (F.Y.); 3 Yunnan Digestive Endoscopy Clinical Medical Center, Department of Gastroenterology, The First People’s Hospital of Yunnan Province, Kunming 650032, China; xunsan@sina.com (L.X.); 15912491520m@sina.com (M.L.); 4 Yunnan Institute of Food and Drug Supervision and Control, Medical Products Administration of Yunnan Province, Kunming 650032, China; jinshumei123321@sina.com (S.J.); chongyi2002@sina.com (C.Y.); 5 School of Pharmacy, Mudanjiang Medical University, Mudanjiang 157011, China; 18367510945@163.com; 6 Guangdong-Hongkong-Macao Joint Laboratory of Respiratory Infectious Disease, State Key Laboratory of Respiratory Disease, Guangzhou Medical University, Guangzhou 510182, China; wjchen@gzhmu.edu.cn; 7 Yunnan Provincial Key Laboratory of Birth Defects and Genetic Diseases, The First People’s Hospital of Yunnan Province, Kunming 650032, China; 8 Yunnan Province Clinical Research Center for Senile Diseases, The First People’s Hospital of Yunnan Province, Kunming 650032, China

**Keywords:** circRNA vaccine, circular RNA, pathogens infection, RNA delivery

## Abstract

Vaccines remain one of the most effective tools in combating infectious diseases, though traditional platforms are constrained by limitations including suboptimal immunogenicity, safety concerns, and manufacturing complexity. Circular RNA (circRNA) vaccines have recently emerged as a novel vaccine modality, demonstrating unique advantages including high stability, low innate immunogenicity, and sustained antigen expression. Although early research has predominantly focused on viral targets, accumulating evidence now supports the application potential of circRNA vaccines against diverse pathogens, particularly antibiotic-resistant bacteria. Through encoding critical antigens or virulence factors, these circRNA vaccines demonstrate capability to induce protective immune responses, presenting a viable alternative to conventional antimicrobial strategies. This review highlights recent advances in circRNA vaccine development, spanning synthetic circularization techniques, delivery approaches, and immunological mechanisms. We emphasize their potential against viral, bacterial, fungal, and parasitic infections, while addressing current challenges and future research directions of this emerging platform. Collectively, these insights underscore circRNA’s multifaceted versatility and its expanding relevance in next-generation vaccine innovation.

## 1. Introduction

Vaccines stand as one of the most impactful achievements in medical history, having drastically reduced the global burden of infectious diseases. Over the past century, conventional vaccine platforms, including live attenuated, inactivated, protein subunit, and polysaccharide conjugate vaccines, have prevented an estimated 2.5 million deaths annually, particularly among infants, through immunization against pathogens such as poliovirus, hepatitis B virus, *Mycobacterium tuberculosis*, *Haemophilus influenzae* type b, rotavirus, and measles [1]. Nevertheless, despite their success, these established approaches are constrained by multiple limitations: the requirement for pathogen cultivation under biosafety containment, lengthy production timelines, potential safety concerns related to whole-pathogen components, and insufficient flexibility in responding to rapidly emerging or mutating pathogens.

The emergence of RNA therapeutics has revolutionized vaccine design, as evidenced by mRNA vaccines demonstrating the potential for rapid development, scalable production, and high efficacy, most notably during the COVID-19 pandemic. Nonetheless, mRNA vaccines’ clinical implementation faces persistent challenges, including thermal stability, cold chain logistics, transient antigen expression, and dose-dependent immunogenicity. These shortcomings have stimulated the pursuit of more resilient RNA platforms, among which circular RNA (circRNA) has gained prominence as a next-generation vaccine candidate capable of overcoming these technical hurdles.

Structurally, circRNAs are covalently closed-loop RNA molecules, lacking both the 5′ cap structure and 3′ poly(A) tail that characterize linear mRNA. This unique topological structure confers intrinsic resistance to exonucleases, enabling extended intracellular stability and sustained antigen expression [2]. Moreover, their terminal integrity mitigates activation of innate immune pathways mediated by Toll-like receptors (TLRs) and RIG-I-like receptors (RLRs), thereby attenuating off-target inflammatory responses [3,4]. These biochemical properties establish circRNA as an exceptionally safer, stable, and longer-acting platform for vaccine development.

Notably, in 2022, Zhang et al. [5] demonstrated that a circRNA vaccine encoding the SARS-CoV-2 spike protein induced a robust humoral response and robust T cell immunity in preclinical models, paving the way for broader applications in infectious disease prevention. Compared to linear mRNA counterparts, circRNA exhibits three key advantages: (1) extended antigen production permitting reduced dosing frequency, (2) enhanced thermostability for ambient storage and transport, and (3) diminished reactogenicity—critical advantages for global vaccine deployment in both high-resource and resource-limited settings. To provide a broader perspective, Table S1 summarizes the distinct advantages and disadvantages of circRNA vaccines compared to other vaccine platforms.

Beyond viral diseases, which have traditionally been the focus of RNA vaccine development, the broader potential of circRNA vaccines is increasingly being recognized. Their ability to encode stable immunogens makes them an attractive platform for combating bacterial pathogens, particularly in the face of rising antimicrobial resistance (AMR), a looming global health crisis. CircRNA vaccines could be tailored to express conserved bacterial antigens or neutralizing antibodies against bacterial toxins, presenting an innovative strategy for tackling multidrug-resistant infections. Additionally, the utility of circRNA vaccines may extend to combating challenging fungal and parasitic infections, such as *Candida albicans* and *Plasmodium falciparum*, for which conventional vaccine platforms have yet to deliver effective solutions [6,7].

Given the accelerating progress in the field, this review provides a timely and comprehensive overview of circRNA vaccine development. First, we elucidate the molecular mechanisms underpinning circRNA biogenesis and synthesis methodologies. Second, we evaluate current advances in pathogen-specific vaccine design, including viruses, bacteria, fungi, and parasites. Third, we identify translational barriers ranging for circularization efficiency to manufacturing scalability, while proposing potential solutions. Finally, we offer insights into emerging innovations and future directions that may guide the clinical translation of circRNA vaccine technologies.

In the face of mounting global demand for immunization solutions that are thermostable, rapidly adaptable, and manufacturing-ready, this analysis seeks to contextualize circRNA vaccine within the evolutionary trajectory of vaccine technology. We particularly underscore their dual applicability in addressing present-day epidemiological threats, while establishing a proactive framework for mitigating future pandemic-scale emergencies.

## 2. Historical Evolution of CircRNA Vaccine Research

The scientific journey of circular RNA (circRNA) begins in the early 1970s with the discovery of subviral pathogens during investigation into potato spindle tuber disease. These entities, later named *viroids*, were found to lack a protein coat, an essential feature distinguishing them from classical viruses—and instead consisted solely of small, circular, single-stranded RNA molecules [8,9]. In 1976, Sanger and colleagues [10] provided the first formal description of viroids as covalently closed circular RNAs. Just a few years later, in 1979, the first physical evidence of circular RNA structures in mammalian cells was obtained via electron microscopy of cytoplasmic fractions isolated from HeLa cells [11].

Initially observed in viral genomes, one of the most remarkable characteristics of circular RNAs lies in their ability to undergo rolling-circle replication, a mechanism that enables efficient amplification and propagation. This characteristic not only highlighted the functional versatility of circular RNAs beyond their structural uniqueness, but also bridges viral molecular strategies with eukaryotic RNA biology. Parallel investigations revealed the endogenous emergence of circRNAs as either functional intermediates or regulatory byproducts during pre-mRNA splicing processes. The field witnessed a paradigm shift in 1991 when Nigro et al. [12] reported the groundbreaking identification of endogenous circRNAs in humans cells, representing a seminal milestone in RNA research. Their work established that these covalently closed RNA molecules are catalyzed by a distinctive back-splicing mechanism, where a downstream splice donor site covalently ligates to an upstream splice acceptor site through non-linear splicing topology.

Building upon these foundational discoveries, subsequent investigations systematically elucidated the regulatory significance of circRNAs within gene expression networks. A pivotal advancement came with the pioneering demonstration that the murine Sry gene—a master regulator of male sex determination—generates functional circRNAs in adult mouse testes [13], providing the first mechanistic link between circular transcripts and developmental biology. The evolutionary conservation of circRNA biogenesis was unequivocally confirmed in 2006 when Jonathan M. et al. identified circular transcripts in *Drosophila melanogaster* [14], establishing circRNAs as phylogenetically conserved RNA species. This field experienced a revolutionary transformation in 2012, when systematic high-throughput RNA sequencing revealed the staggering prevalence of circRNAs across eukaryotic species, with genomic analyses demonstrating that circular isoforms constitute the predominant transcript variant for numerous human genes [15].

The mechanistic underpinnings of circRNA functionality gained substantial clarity in 2013, when seminal research demonstrated their capacity to function as potent endogenous sponges for microRNAs (miRNAs), thereby establishing a novel competing endogenous RNA (ceRNA) mechanistic framework for post-transcriptional regulation [16]. This discovery fundamentally reshaped our understanding of non-coding RNA interactomes. A paradigm-shifting breakthrough emerged in 2019, when rigorous characterization revealed that engineered circRNAs exhibit inherently low immunogenicity—a critical advantage for pharmacotherapeutic development. Importantly, epigenetically modified circRNAs bearing m6A (N6-methyladenosine) moieties were shown to engage dual mechanisms of immune evasion, intrinsic immunogenicity suppression combined with active inhibition of innate immune activation pathways [17,18], thereby establishing an optimized safety paradigm for systemic administration.

**Figure 1:**
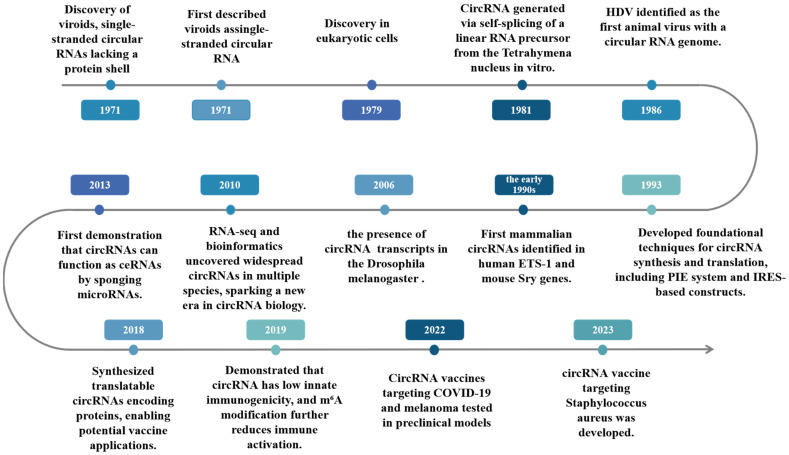
Key development timeline of circRNA vaccines. Timeline of key milestones in circRNA discovery, functional characterization, and vaccine development. Timeline illustrating major advances in circRNA biology, from early discoveries of circular RNAs to their functional characterization and recent application in vaccine development.

The year 2022 witnessed transformative progress in synthetic circRNA engineering through rational optimization of untranslated region (UTR) architecture flanking open reading frames, which achieved unprecedented enhancement in translational fidelity and output [19]. Cumulatively, these methodological breakthroughs have enabled industrial-scale production of artificial circRNAs with pharmaceutical-grade precision, positioning them as multifunctional platforms for molecular interventions spanning epigenetic modulation, de novo protein synthesis, and vaccinology. The unique triad of biochemical stability, programmable translational capacity, and stealth immunogenicity propels engineered circRNAs to the forefront of next-generation therapeutic development, particularly in vaccine innovation, where they enable dual-mode immune modulation through either direct antigen presentation or precise regulation of immune checkpoints. Proof of concept was spectacularly demonstrated by a circRNA vaccine expressing the SARS-CoV-2 receptor-binding domain (RBD) supramolecular trimer—this construct elicited broad-spectrum neutralizing antibodies and durable polyfunctional T cell responses in murine and primate models [5], validating the clinical-translational potential of this RNA nanotechnology (Figure 1).

The therapeutic scope of circRNA technology has expanded into onco-immunology, transcending its original applications in infectious disease prophylaxis. Emerging evidence illuminates a dual functionality paradigm wherein noncoding circRNAs orchestrate tumor-immune crosstalk through epigenetic modulation, independently of their protein-coding capacity. A prime exemplar is circRERE, which operates as a master regulatory node within the competing endogenous RNA (ceRNA) axis by sequestering miR-6837-3p, thereby de-repressing MAVS expression and perpetuating a self-reinforcing cycle of type I interferon (IFN) signaling amplification [20]. This molecular circuitry not only potentiates antigen-presenting cell activation, but also reprograms the tumor microenvironment toward an immunogenic state. Preclinical validation via adeno-associated virus (AAV)-mediated circRERE delivery, when synergized with anti-PD-1 checkpoint blockade, achieves remarkable tumor regression in colorectal carcinoma models, demonstrating the feasibility of circRNA-based combination immunotherapy regimens for overcoming therapeutic resistance.

At the molecular level, circRNAs represent a specialized subclass of long non-coding RNAs (lncRNAs) defined by their covalently closed, topologically constrained circular architecture—a structural hallmark that eliminates the canonical 5′ cap structures and 3′ poly(A) tails characteristic of linear mRNAs. While the majority of circRNAs are canonically processed through back-splicing of exonic sequences within precursor mRNAs, alternative origins include intronic lariat intermediates and untranslated region (UTR)-derived circularization events, particularly those involving 3′UTR or 5′UTR sequences [21]. This structural resilience confers remarkable nuclease resistance against 3′–5′ exonuclease activity (e.g., RNase R), thereby granting substantially extended intracellular persistence relative to their linear counterparts. Such biostability not only underpins their functional longevity, but also positions circRNAs as biomarkers with enhanced diagnostic utility in clinical settings.

Collectively, the metamorphosis of circRNA investigation—spanning from primordial viroid characterization through endogenous regulatory network elucidation to contemporary molecular vaccine engineering—epitomizes the transformative capacity of this topologically constrained nucleic acid species in bridging fundamental RNA biology with clinical translation. Propelled by convergent advances in synthetic nucleic acid architecture and targeted delivery vector development, the circRNA vaccine paradigm is poised to revolutionize both prophylactic and therapeutic strategies against oncogenic pathologies and high-risk pathogens within the next decade, ushering in a new epoch of programmable RNA therapeutics with precise immunomodulatory control.

## 3. Mechanism of Immune Activation by circRNA Vaccines

CircRNA vaccines function by engaging both the innate and adaptive arms of the immune system. Upon administration, typically via lipid nanoparticle (LNP) formulations, circRNAs are internalized by cells through endocytosis. The acidic environment within endosomes facilitates LNP protonation and membrane fusion, enabling cytosolic release of the circRNA payload [22]. Once in the cytoplasm, circRNAs utilize internal ribosome entry sites (IRESs) or m^6^A-mediated initiation to drive cap-independent translation of antigenic proteins [18]. These intracellular antigens are subsequently processed by proteasomes into peptides, which are presented on MHC class I molecules to activate CD8^+^ cytotoxic T cells. Simultaneously, full-length antigenic proteins secreted into the extracellular space can be recognized directly by B cell receptors or taken up by antigen-presenting cells (APCs), processed via lysosomal degradation, and presented to CD4^+^ helper T cells via MHC class II. This cascade activates B cell maturation and antibody production, establishing robust humoral immunity [23].

**Figure 2:**
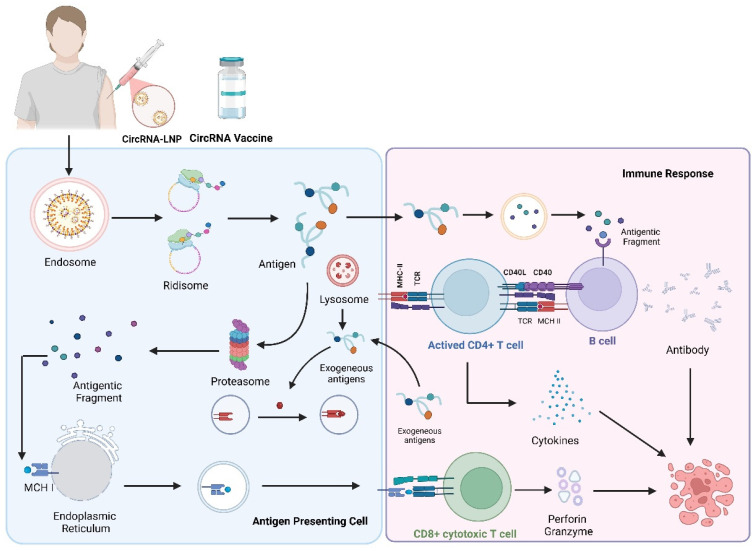
Molecular mechanism of circRNA vaccine activity in vivo. CircRNA vaccines delivered via LNPs are taken up by antigen-presenting cells (APCs) and translated into antigens by ribosomes. Antigens are processed by the proteasome and presented via MHC I to activate CD8^+^ T cells, which secrete cytokines and cytolytic molecules to initiate cellular immunity. Antigens can also be presented via MHC II, activating CD4^+^ T cells that assist B cells in producing neutralizing antibodies, thereby promoting humoral responses.

Importantly, circRNAs also interact with cytosolic and endosomal pattern recognition receptors (PRRs) that monitor for foreign nucleic acids. Structural features such as double-stranded regions, 5′-triphosphate ends, and unmodified nucleotides can activate innate sensors including RIG-I, protein kinase R (PKR), and toll-like receptor 3 (TLR3). RIG-I activation triggers IRF3/7-dependent transcription of type I interferons [24], while PKR engagement leads to phosphorylation of eIF2α and translational suppression [25]. TLR3, primarily located in endosomal compartments, recognizes dsRNA motifs and further amplifies interferon-mediated responses [26]. The magnitude and quality of this innate stimulation depend heavily on circRNA sequence composition, circularization method, and purification stringency. For example, circRNAs containing residual intronic sequences or lacking nucleotide modifications tend to provoke stronger innate responses, whereas sequence-optimized, chemically modified circRNAs exhibit reduced immunogenicity [27] (Figure 2).

## 4. Synthesis Process of circRNA

### 4.1. Precursor Linear RNA Synthesis

The engineering of circRNA begins with the precision synthesis of linear RNA precursors that must possess three critical attributes: (1) defined terminal functional groups for cyclization chemistry, (2) optimized sequence architecture to prevent secondary structure interference, and (3) sufficient molecular length to maintain functional integrity post-circularization. Chemically synthesized oligonucleotides offer unparalleled control over 5′-monophosphate end-group incorporation during solid-phase assembly, thereby simplifying downstream enzymatic or chemical ligation protocols [23]. Nevertheless, this methodology faces inherent limitations in scaling for sequences exceeding 70 nucleotides (nt), where chromatographic purification escalates production costs while compromising synthetic yields below pharmacologically viable thresholds [28]. Moreover, non-physiological phosphodiester linkages (e.g., 2′,5′-bond formation) frequently arise from carbodiimide-mediated condensation using 1-ethyl-3-(3-dimethylaminopropyl)carbodiimide (EDC) or cyanogen bromide, introducing structural artifacts that may compromise biological activity.

Conversely, bacteriophage-polymerase-driven in vitro transcription (IVT) remains the gold-standard methodology for large-scale production of longer RNA precursors (typically 100–5000 nt). In this template-directed process, linearized plasmid DNA encoding target sequences flanked by promoter elements (e.g., T7 or SP6) undergoes high-fidelity RNA polymerization in the presence of ribonucleotide triphosphates (NTPs) and magnesium cofactors [29]. This system achieves milligram-scale yields of pharmaceutical-grade RNA with native 5′-triphosphate and 3′-hydroxyl termini—structural features that necessitate post-transcriptional phosphatase treatment (when 5′-monophosphate ends are required for splint ligation approaches) [30]. The IVT platform strikes an optimal balance among sequence flexibility, product length capacity, and manufacturing scalability, making it indispensable for therapeutic circRNA development.

In physiological systems, circRNA biogenesis is orchestrated through evolutionarily conserved back-splicing mechanisms—including lariat-driven circularization, exon skipping events, intronic complementary sequence pairing, and trans-factor-mediated splicing—wherein the 5′ splice donor site covalently ligates to a downstream 3′ splice acceptor site, thereby establishing canonical 3′,5′-phosphodiester bonds [23]. In stark contrast, in vitro circularization necessitates exogenous catalytic systems (categorized as chemical conjugants, enzymatic ligases, or ribozyme-based platforms) to mediate terminal juxtaposition and phosphodiester bond formation. The subsequent subsections will methodically analyze these three distinct in vitro circularization strategies, with particular emphasis on their mechanistic principles, operational efficiencies, and technical limitations.

#### 4.1.1. Chemical Synthesis

Chemoselective ligation strategies harness the nucleophilic reactivity of 5′-phosphate moieties toward 3′-hydroxyl groups to establish phosphodiester bonds under bioorthogonal reaction conditions. Bifunctional coupling agents such as 1-ethyl-3-(3-dimethylaminopropyl)carbodiimide (EDC) or cyanogen bromide mediate phosphoryl activation, thereby driving entropy-driven intramolecular condensation [28]. Notwithstanding conceptual elegance, the ligation efficiency exhibits an inverse correlation with precursor length (>50 nucleotides) due to steric hindrance and kinetic trapping of terminal nucleotides. A critical limitation arises from noncanonical 2′,5′-phosphodiester bond formation (~15–30% of total linkages), which induces structural isomerization and functional impairment through altered RNA folding thermodynamics [23]. The necessity for multi-step HPLC purification to eliminate unreacted substrates and hydrolytic byproducts escalates production costs beyond pharmaceutically viable thresholds (<5% isolated yield for 100 nt constructs), rendering chemical methods largely obsolete for therapeutic-grade circRNA synthesis. Consequently, these approaches are predominantly employed for short functional motifs (<70 nt) in proof-of-concept studies, with enzymatic and ribozyme-mediated circularization emerging as the preferred modalities in translational research (Table S2).

#### 4.1.2. Enzymatic Synthesis

Enzymatic ligation provides a reliable route to native 3′,5′-phosphodiester bonds without the need for harsh chemicals. Three ligases are most commonly employed: T4 RNA ligase 1 (Rnl1), T4 DNA ligase, and T4 RNA ligase 2 (Rnl2). Rnl1 catalyzes the nucleophilic attack of a 3′-hydroxyl group on an activated 5′-phosphate, achieving efficient circularization of single-stranded RNAs up to approximately 500 nucleotides in length [31]. However, its efficiency diminishes with longer transcripts and higher precursor concentrations, where intermolecular oligomerization becomes a significant side reaction [27]. T4 DNA ligase requires a complementary DNA splint to align RNA ends precisely, adding a design and annealing step but improving specificity. Rnl2, which repairs diverse RNA termini in vivo, can ligate both single- and double-stranded junctions when guided by an RNA splint, though it too suffers reduced activity on longer substrates and similar side reactions [32]. A key advantage of enzymatic ligation is that it yields circRNAs free of extraneous sequences, thereby minimizing activation of innate immune sensors. The choice among Rnl1, DNA ligase, and Rnl2 depends on precursor length and secondary structure: Rnl1 and DNA ligase are optimal for unstructured or splinted RNAs, whereas Rnl2 is preferred when the ligation site resides within a duplex region. Future efforts should focus on engineering ligases with enhanced processivity, optimizing reaction conditions (for example, including molecular crowding agents), and developing strategies to suppress undesired end-joining.

#### 4.1.3. Ribozyme-Mediated Circularization

Autocatalytic RNA motifs enable protein-independent synthesis of precisely defined circRNAs through self-processing intronic elements. The permuted intron–exon (PIE) system—derived from group I intron ribozymes—has emerged as the gold-standard platform for scarless RNA circularization. This system executes two sequential transesterification reactions catalyzed by guanine-coordinated Mg²^+^ clusters: (1) 3′-OH activation via GTP hydrolysis and (2) concerted exon ligation with intronic lariat excision, yielding covalently closed RNA circles exhibiting atomic-level backbone integrity [33].

Engineered circular RNA regulators (ECRRs) amplify this process through dimeric assembly at precursor termini, enforcing allosteric control over intronic scaffold folding kinetics to achieve >95% circularization efficiency [34]. Group II introns similarly mediate high-fidelity circularization without sequence remnants, though their in vitro catalytic trajectory remains mechanistically obscure [23]. Hairpin ribozymes (HPRs) enable rolling circle amplification from single-stranded DNA templates, yet the dynamic equilibrium between site-specific cleavage and ligation (Kd ≈ 10^−3^ M^−1^) combined with persistent ribozyme motifs in final products severely constrains pharmaceutical scalability [35].

The PIE methodology currently delivers unparalleled sequence fidelity (error rate <0.01%), multi-gram scalability, and complete scarless architecture, solidifying its position as the premier technology for industrial-scale vaccine-grade circRNA production.

## 5. Delivery Strategies

Effective delivery of circular RNA vaccines requires carriers that protect the RNA from degradation, facilitate cellular uptake, avoid premature immune clearance, and release their cargo into the appropriate intracellular compartment. The principal platforms under investigation include lipid nanoparticles, exosomes, and a variety of emerging physical and biological methods, each with distinct advantages and limitations.

### 5.1. Lipid Nanoparticles (LNPs)

Lipid nanoparticles represent the leading delivery system for RNA vaccines. A typical formulation comprises an ionizable lipid, a phospholipid, cholesterol, and a polyethylene glycol–lipid (PEG–lipid) [36]. At physiological pH (approximately 7.4), the ionizable lipid remains neutral, which minimizes interactions with serum proteins and enhances circulatory stability. After endocytosis, the lower pH of the endosome (approximately 5.0 to 6.0) protonates the ionizable lipid, promoting disruption of the endosomal membrane and release of circRNA into the cytosol [37]. PEG–lipids confer steric stabilization, reduce opsonization by serum proteins, and prolong systemic circulation, but excessive PEG content may hinder interactions with target cells and reduce transfection efficiency [38,39]. Phospholipids such as DSPC and DPPC contribute to membrane integrity and further facilitate endosomal escape [38,40].

Manufacturing techniques based on microfluidic mixing produce LNPs with uniform size and high encapsulation efficiency [41]. Using this approach, researchers formulated multi-armed ionizable lipids and compared their performance to clinically approved formulations such as MC-3 and SM-102. Intramuscular administration of LNPs carrying circRNA encoding ovalbumin in mice elicited elevated levels of proinflammatory cytokines without detectable organ toxicity at 24 h post injection [42]. Charge-altering releasable transporters (CARTs) have also been developed; these molecules bind RNA through transient cationic charges and then degrade into neutral products, sustaining circRNA expression for up to 96 h after intravenous delivery [19,43]. In another study, multi-armed ionizable lipids combined with a novel ionizable lipid in AX4-LNPs accelerated degradation in splenic cells, thereby enhancing circRNA release and promoting a local proinflammatory environment that activated cytotoxic T lymphocytes while minimizing tissue damage [5,44]. High-throughput combinatorial screening further identified an H1L1A1B3 formulation that, when loaded with circRNA encoding interleukin-12 and delivered intratumorally, induced significant tumor regression in a Lewis lung carcinoma model, accompanied by increased infiltration of CD45-positive leukocytes and CD8-positive T cells [45]. Intramuscular injection of circRNA encoding the SARS-CoV-2 receptor-binding domain in FDA-approved LNPs generated robust antigen-specific IgG responses in both mice and rhesus macaques [5].

Despite these successes, systemic or local administration of LNPs often results in accumulation within the liver, which can provoke hepatotoxicity. Structural optimization of lipid headgroups, linkers, and hydrophobic tails led to the design of the 113-O12B LNP, which preferentially delivers RNA to lymph nodes and reduces hepatic exposure, thereby mitigating the risk of immune-mediated liver injury [46]. Continued innovation in lipid chemistry and formulation techniques will be essential to improve targeting specificity and safety.

### 5.2. Exosome-Based Delivery

Exosomes (40–60 nm) represent evolutionarily optimized extracellular vesicles endogenously secreted by eukaryotic cells, exhibiting intrinsic biocompatibility and negligible immunogenicity [47]. Their phospholipid bilayers are natively decorated with integrins and tetraspanins (CD63/CD81), enabling stealth traversal through mononuclear phagocyte systems while maintaining ligand–receptor targeting specificity [48]. Li et al. pioneered mitochondriotropic exosome engineering by co-encapsulating circRNA-mSCAR with poly-D-lysine-triphenylphosphonium (PDK-TPP) conjugates, achieving ~80% mitochondrial colocalization in primary macrophages that drove IL-10-mediated M2 polarization and rescued 70% survival in murine sepsis models [49]. Notably, RVG-peptide-functionalized exosomes demonstrated blood–brain barrier penetration (>10-fold higher CNS accumulation), delivering circSCMH1 to peri-infarct cortex with >50% neurite regeneration in non-human primate stroke models [50].

Key challenges for exosome-based delivery include scalable isolation and purification, efficient cargo loading, and batch-to-batch consistency. Addressing these issues through standardized manufacturing protocols, advanced loading technologies (for example, electroporation or membrane permeabilization), and surface engineering to enhance tissue tropism will be critical for clinical translation.

### 5.3. Emerging Delivery Platforms

Electroporation-based systems employ millisecond-scale electrostatic pulses (8–10 kV/cm) to induce transient nanopores (4–6 nm) in plasma membranes, enabling direct cytoplasmic delivery of nucleic acids [51]. A breakthrough nanochannel electroinjection platform achieved >70% transfection efficiency for plasmid DNA, mRNA, and circRNA in human dendritic cells while maintaining >85% cell viability [52]. Notably, in primary murine bone marrow-derived DCs, this system delivered circRNA with 68.3% efficiency without inducing premature maturation or apoptotic cascade activation. However, irreversible membrane damage (<15% cell death) and batch-dependent variability constrain clinical scalability.

Viral vector engineering has enabled sustained circRNA expression through genomic integration of circRNA-encoding minicircle DNA. Adeno-associated virus (AAV) vectors encoding self-splicing circular guide RNAs demonstrated >90% RNA editing persistence for 6 months across neural/hematopoietic lineages [53]. Virus-like particles (VLPs) provide non-integrative alternatives, exemplified by HCV core protein–VLPs inducing neutralizing antibody titers >1:1024 in prime-boost regimens [54]. Persistent concerns regarding insertional oncogenesis (~0.1% frequency) and vector-directed immunogenicity mandate rigorous safety profiling.

Localized delivery strategies show particular promise for oncoimmunotherapy. Intratumoral administration of circRNA vaccines in subcutaneous NSCLC, melanoma, and CRC xenografts upregulated IFN-γ (>eight-fold) and Granzyme B (>five-fold), reprogramming immunosuppressive microenvironments toward CD8^+^ T cell-dominated anti-tumor immunity [55]. Poly(lactic-co-glycolic acid) (PLGA) nanoparticles engineered with pH-sensitive coatings achieved 72 h sustained release with <5% systemic exposure, significantly enhancing tumor-infiltrating lymphocyte (TIL) activation.

**Figure 3:**
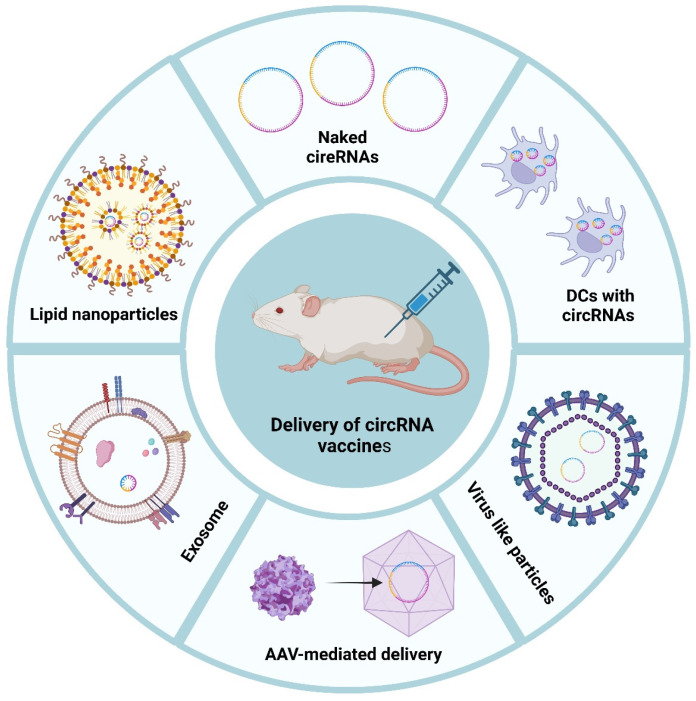
Different delivery system of circRNA. Schematic illustration of various delivery strategies for circRNA-based vaccines. Multiple approaches have been explored to enhance the delivery efficiency and immunogenicity of circular RNA (circRNA) vaccines, including (1) naked circRNAs, (2) dendritic cells (DCs) loaded with circRNAs, (3) virus-like particles (VLPs), (4) adeno-associated virus (AAV)-mediated delivery, (5) exosomes, and (6) lipid nanoparticles (LNPs).

Research on circRNA delivery systems continues to evolve. Future efforts must focus on refining carrier design to improve targeting specificity, scalability, and in vivo stability. Innovations in nanoparticle composition, RNA chemical modification, and combination therapies with immune modulators or gene editing technologies promise to expand the therapeutic potential of circRNA vaccines for infectious diseases, cancer, and genetic disorders (Figure 3).

## 6. Current Research and Development in CircRNA Vaccines for Pathogen-Targeted Applications

### 6.1. CircRNA Vaccines Against Viral Pathogens

As our understanding of circular RNAs advances, it has become clear that these molecules play multifaceted roles in viral infection and host defense. Host-derived circRNAs have been detected in infections caused by hepatitis viruses, SARS-CoV-2, influenza virus, herpesviruses, and Epstein–Barr virus, among others [56]. In some cases, viruses exploit specific circRNAs to enhance their replication and pathogenicity, whereas other circRNAs act as innate immune modulators, limiting viral spread and shaping cytokine responses [57].

#### 6.1.1. SARS-CoV-2

CircRNA biogenesis is generally thought to depend on RNA polymerase II transcription and the back-splicing of pre-mRNA. Yang and colleagues demonstrated that beta coronaviruses generate abundant circRNAs even when canonical host transcription and splicing are inhibited, indicating an alternative mechanism of circularization [58]. In SARS-CoV-2–infected cells, the balance of pro- and anti-inflammatory cytokines is strongly influenced by noncoding RNAs. In particular, hsa_circ_0000479 is upregulated in the blood of COVID-19 patients relative to healthy controls, concomitant with increased expression of RIG-I and interleukin-6 (IL-6) and decreased levels of miR-149-5p [59]. Mechanistic studies reveal that hsa_circ_0000479 sequesters miR-149-5p, thereby derepressing RIG-I and driving IL-6 production. This circRNA–miRNA–mRNA regulatory axis appears to be a critical modulator of the cytokine milieu during SARS-CoV-2 infection [60].

Beyond their endogenous regulatory functions, circRNAs can be engineered to serve as antigen-encoding vaccines or therapeutic protein vectors. Qu et al. [5] developed a circRNA vaccine encoding a trimeric receptor-binding domain (RBD) of the SARS-CoV-2 spike protein. Following intramuscular administration in mice and rhesus macaques, this formulation elicited high titers of neutralizing antibodies and robust T cell responses, conferring protection against viral challenge. In parallel, circRNAs encoding multivalent nanobodies specific for the SARS-CoV-2 spike protein have demonstrated potent neutralization of pseudovirus infection [5,27,61,62,63]. This platform further offers the possibility of in vivo expression of therapeutic antibodies such as anti-PD-1 or anti-PD-L1, and even intracellular targeting of oncogenic proteins like TP53 and KRAS, thereby overcoming the limitations of conventional antibody therapies [64,65,66,67]. Collectively, these studies establish circRNA as a versatile and potent modality for both vaccination and protein-based therapeutics against SARS-CoV-2.

#### 6.1.2. Influenza

The success of mRNA vaccines against influenza A virus (IAV) has underscored the potential of RNA-based platforms. For example, an mRNA vaccine encoding the full-length matrix-2 ion channel, hemagglutinin stalk domain, neuraminidase, and nucleoprotein conferred broad protection in animal models [68]. Recent work has begun to elucidate the roles of endogenous circRNAs in IAV infection. Circ-GATAD2A promotes viral replication by inhibiting VPS34-dependent autophagy, a host process that normally restricts IAV [69]. Similarly, circRNA_0050463 sponges miR-33b-5p, thereby upregulating eukaryotic translation elongation factor 1 alpha 1 and facilitating viral protein synthesis [70]. In contrast, the circRNA designated AIVR enhances interferon-β production in infected A549 cells by acting as a decoy for specific miRNAs and upregulating CREBBP expression [71]. Additional circRNAs such as circMerTK have been shown to suppress innate antiviral signaling and thereby support viral propagation by modulating interferon-β pathways [72]. Conversely, circVAMP3 interferes with assembly of the viral ribonucleoprotein complex by binding NP and NS1 proteins and relieving NS1-mediated inhibition of RIG-I and TRIM25, resulting in enhanced interferon responses and restricted viral replication [73].

Together, these discoveries highlight the dual potential of circRNAs in influenza: as targets whose modulation may enhance host defense and as templates for RNA vaccines that encode viral antigens or immune-modulatory factors. By harnessing the unique stability and functional versatility of circRNAs, it may be possible to develop next-generation influenza vaccines that elicit broader and more durable protection than existing approaches.

#### 6.1.3. Zika Virus

Zika virus (ZIKV) is a mosquito-borne flavivirus that belongs to the Flaviviridae family and is closely related to dengue virus (DENV). The immune cross-reactivity between ZIKV and DENV necessitates careful consideration in vaccine development in order to avoid antibody-dependent enhancement (ADE) [74]. Vaccines that include the full-length E protein or a combination of the E protein with the prM protein may elicit non-neutralizing, cross-reactive antibodies. Therefore, targeting domain III of the E protein (EDIII) is preferred because it induces a more specific immune response against both ZIKV and DENV, while effectively reducing the risk of ADE [75]. However, the immunogenicity of EDIII is intrinsically suboptimal, which may require repeated doses to achieve sufficient protective immunity. For instance, Dengvaxia, a tetravalent live-attenuated vaccine, induces DENV4-specific neutralizing antibodies and cross-reactive antibodies against DENV1–3, but it increases the risk of severe dengue in DENV-naïve individuals [76]. CircRNA platforms have emerged as promising candidates because of their inherent stability, independence from a 5′ cap, and ability to drive continuous protein expression. A recent study developed a circRNA vaccine targeting ZIKV by employing constructs encoding an EDIII-Fc fusion and NS1. Administration of EDIII-Fc alone provided partial protection in mice, whereas co-delivery with NS1 enhanced efficacy. A single dose of the optimized circRNA vaccine conferred durable protection without inducing ADE of dengue virus infection. These results highlight the potential of circRNA vaccines for safe and effective ZIKV prevention [77].

#### 6.1.4. Hepatitis Virus

Hepatitis B virus (HBV) is a partially double-stranded DNA virus with a genome of approximately 3200 base pairs. It is one of the most prevalent chronic viral infections and is associated with severe liver diseases, including cirrhosis and hepatocellular carcinoma. Recent investigations have identified several circRNA/miRNA/mRNA pathways that are differentially expressed in HBV-infected cells [78]. In particular, hsa_circ_0005389 and hsa_circ_0000038 have been linked to HBV replication; these circRNAs modulate antiviral immunity and HBV gene expression by affecting interferon-α-mediated pathways and by targeting key viral enhancers and transcription factors. For example, hsa_circ_0005389 enhances antiviral immunity by regulating IRF7 [79], while hsa_circ_0000038 inhibits HBV replication through interactions with miR-370 and miR-939, which target the HBV enhancer and core promoter regions [80,81]. Moreover, the Circular RNA Interactome database has indicated that hsa_circ_0000976, which contains a binding site for hsa-miR-486-3p, may influence chemokine- and cytokine-mediated immune responses in infected hepatocytes [21]. Additional studies have shown that circ_00004812 is upregulated in HBV-infected cells; its knockdown results in increased expression of interferon-α and interferon-β, suggesting a role in regulating antiviral responses during HBV infection [82]. Interestingly, circRNAs may also serve as therapeutic agents. An artificial circRNA designed as a microRNA sponge was able to disrupt hepatitis C virus replication by sequestering miR-122, a strategy similar to the mechanism of action of Miravirsen [83]. This approach suggests that a similar circRNA-based strategy could be explored for HBV.

#### 6.1.5. Other Viruses

**Figure 4:**
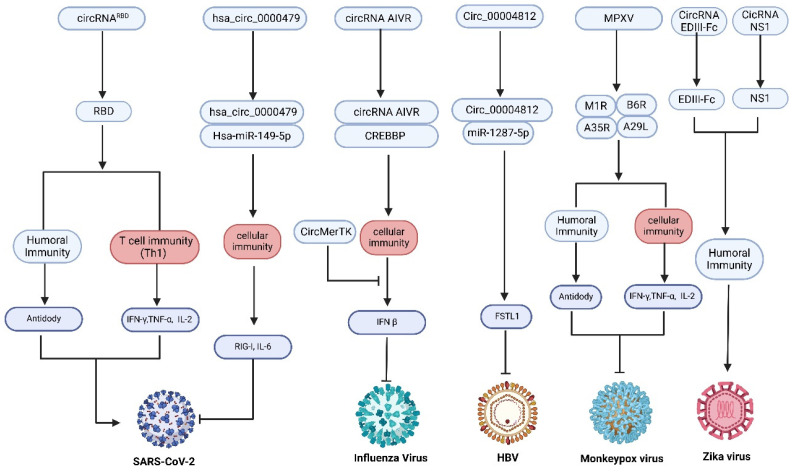
Immune activation by circRNA vaccines in antiviral applications. CircRNA vaccines activate antiviral immune responses against a range of viruses, including SARS-CoV-2, influenza, HBV, monkeypox, and Zika virus. Through circRNA–miRNA–mRNA regulatory networks, they modulate immune-related gene expression, thereby initiating both humoral and cellular immunity. This immune activation is characterized by increased cytokine production, CD4^+^/CD8^+^ T cell responses, and the generation of neutralizing antibodies, collectively contributing to effective antiviral defense.

CircRNA vaccines hold potential for applications beyond the previously discussed pathogens. RNA-based vaccines have already been developed for several viral infections, providing proof of concept for this approach. For example, an mRNA vaccine encoding a combination of HIV-1 Gag, Pol, and Nef proteins has been developed to protect against HIV infection [84]. Additionally, mRNA vaccines encoding glycoproteins gC, gD, and gE for herpes simplex virus (HSV) have demonstrated strong protection against HSV-1, eliciting both neutralizing antibodies and CD8^+^ T cell responses, with an HSV-2-based vaccine showing cross-protection [85]. Zhang and colleagues reported that Bombyx mori cypovirus (BmCPV), a double-stranded RNA virus, generates viral circRNAs, including vcircRNA_000048, which encodes a micropeptide (vsp21) that attenuates viral replication in an internal ribosome entry site (IRES)-dependent manner [86]. This finding provides new insights into viral regulation and host–virus interactions. In addition, Zuiani et al. [87] demonstrated that BNT166a, a multivalent mRNA vaccine targeting monkeypox virus, achieved 100% efficacy in preventing death and reducing lesion severity in cynomolgus macaques challenged with a lethal strain of monkeypox virus. Building on this concept, a recent study developed a multivalent circRNA vaccine against monkeypox virus that elicited robust humoral and cellular immune responses in mice and provided effective protection against viral challenge [88]. Given their improved stability and protein expression profiles, circRNAs represent a promising alternative to mRNA for multivalent vaccine development. Further investigation is warranted to evaluate their advantages and translational potential in clinical settings (Figure 4).

### 6.2. CircRNA Vaccines Against Bacterial Pathogens

Bacterial, parasitic, and fungal infections continue to pose serious public health challenges, particularly due to the emergence of drug-resistant strains. Resistant bacterial infections, in particular, are associated with poor clinical outcomes, increased medication usage, and higher medical expenditures. Hence, the development of effective vaccines remains a priority for early intervention and prevention.

CircRNA vaccines offer significant potential in the fight against bacterial infections due to their high stability and ability to elicit both humoral and cellular immune responses. Although antibacterial mRNA vaccines have demonstrated protective efficacy in preclinical studies against group A and group B streptococcus (GAS and GBS, respectively) [89], they are still undergoing early-stage evaluation. In contrast to conventional bacterial vaccines—which often face challenges such as immune evasion and strain variability—circRNA vaccines provide a flexible platform capable of encoding multiple antigens, thereby enhancing cross-protection against diverse bacterial strains. Thus, circRNA-based vaccine development represents a promising strategy for both therapeutic and prophylactic interventions against bacterial pathogens.

#### 6.2.1. *Staphylococcus aureus*

*Staphylococcus aureus* is a versatile pathogen implicated in a wide range of clinical conditions, from superficial skin and soft tissue infections to severe systemic diseases, including bacteremia, pneumonia, osteoarticular infections, and septic shock [90]. Of particular concern is methicillin-resistant *S. aureus* (MRSA), which, though initially confined to healthcare settings, has emerged as a major cause of community-acquired infections [90].

Despite the development of recombinant protein vaccines against *S. aureus*, their limited ability to elicit robust T cell responses significantly diminishes their protective capacity [91]. In contrast, RNA-based vaccines offer the ability to stimulate both arms of the adaptive immune response [92]. Among these, circRNA vaccines stand out for their enhanced stability and prolonged antigen expression. Inspired by their success in viral vaccine platforms, circRNA technologies are now being applied to bacterial targets. One notable approach involved designing a multi-epitope circRNA vaccine encoding six conserved antigens (FnBA, HlgA, HlgB, ISDB, SpA, and Eno), providing broad protection against seven clinically relevant MRSA strains from both community and hospital settings [7]. This multi-targeted strategy highlights the potential of circRNA-based vaccines in combating multidrug-resistant *S. aureus*.

Beyond antigen delivery, circRNAs have also been implicated in regulating host immune responses during *S. aureus* infections. For instance, Li et al. [93] developed an osteoblast infection model and identified dysregulation of circRNAs, such as hsa_circ_0002483, which modulates PTK2 signaling by sponging hsa-miR-6886-5p. Similarly, circSyk was upregulated in infected osteoclasts, promoting bone destruction via the circSyk/miR-5106/Sik3 axis [94]. These findings suggest that circRNAs may influence infection persistence and recurrence by modulating intracellular signaling and autophagy.

#### 6.2.2. *Mycobacterium tuberculosis*

*Mycobacterium tuberculosis* (Mtb) infection initiates a complex host immune response, with macrophages serving as the first line of defense. However, Mtb has developed sophisticated strategies to evade macrophage-mediated killing, contributing to latent or active tuberculosis. Recent evidence highlights the role of circRNAs as key modulators of macrophage functions during Mtb infection. Multiple circRNAs have been identified as potential biomarkers or immune regulators in active pulmonary TB. These included hsa_circ_005836, hsa_circ_0001380, circRNA_051239, circRNA_029965, hsa_circ_0028883, and circRNA_404022 [95].

CircRNAs have been shown to influence macrophage autophagy, apoptosis, and polarization, thereby affecting Mtb clearance. For example, hsa_circ_0045474, downregulated in TB patients, promotes autophagy through the miR-582-5p/TNKS2 axis [96], suggesting it as a candidate for enhancing macrophage-mediated bacterial clearance. Luo et al. [97] demonstrated that circTRAPPC6B promotes autophagy by suppressing miR-874-3p-mediated inhibition of ATG16L1. Likewise, hsa_circ_0003528 modulates macrophage polarization towards the M1 phenotype via interactions with miR-324-5p, miR-224-5p, and miR-488-5p, ultimately promoting an antibacterial state and reducing immune evasion [98].

While the BCG vaccine remains effective in children, its protective efficacy against adult pulmonary TB is limited. Several new vaccine candidates—including H56:IC31, M72:AS01E, BNT164a1/BNT164b1, ChAdOx1.85A/MVA85A, and MTBVAC—are under clinical evaluation [99]. CircRNA-based vaccines present a novel alternative, capable of regulating immune evasion mechanisms and boosting autophagic responses. For instance, circAGFG1 and circTRAPPC6B have shown potential in enhancing autophagy and suppressing Mtb growth [97,100]. Moreover, high-throughput sequencing has identified additional circRNAs, such as hsa_circ_0007919 and hsa_circ_0002419, that interact with immune-related pathways [101], offering new targets for vaccine development.

Advancements in delivery platforms, such as lipid nanoparticles (LNPs), further enhance the potential of circRNA vaccines by improving antigen expression and intracellular stability. These approaches may significantly amplify macrophage function, leading to more effective Mtb clearance and immune protection.

#### 6.2.3. *Escherichia coli*

*Escherichia coli* (*E. coli*), particularly multidrug-resistant strains, pose substantial health risks due to increasing resistance to β-lactams and fluoroquinolones. *E. coli* is a leading cause of bacterial meningitis—a condition marked by high morbidity, mortality, and long-term neurological deficits. CircRNAs have emerged as important regulators in bacterial meningitis, with differential expression patterns observed in infection models. Downregulation of circRNAs such as circRNA_6577, circRNA_7725, circRNA_0309, circRNA_2125, and circRNA_3832 and upregulation of circRNA_7711 have been implicated in the host response through complex circRNA–miRNA–mRNA networks [102].

Additionally, specific circRNAs—including hg38_circ_0027134, hg38_circ_0032477, hg38_circ_0002276, and hg38_circ_0031043—modulate blood–brain barrier permeability, directly influencing disease progression [103]. Other circRNAs, such as hg38_circ_0008980 and hg38_circ_0001582, participate in protein regulation and metabolism. Notably, a recent study introduced a simple and efficient method for one-step circRNA synthesis using *E. coli*, offering improvements in cost, yield, and downstream applications for protein translation and RNA interference.

Due to the high variability of *E. coli* serotypes, particularly between pathogenic and commensal strains, vaccine development must prioritize antigens specific to pathogenic types. Surface-exposed antigens are ideal vaccine targets [104]. Additionally, circRNAs may help preserve intestinal microbiota homeostasis. For instance, hsa_circ_0001021 regulates epithelial barrier integrity in ulcerative colitis by sponging miR-224-5p [105], suggesting potential for selective immune modulation. Future circRNA vaccine strategies should focus on preserving microbial balance while targeting pathogenic *E. coli*, especially in mucosal vaccine formulations.

#### 6.2.4. Other Bacterial Pathogens

Emerging evidence supports the regulatory roles of circRNAs in host responses to various bacterial infections beyond the major human pathogens. In *Salmonella enteritidis*-infected duck granulosa cells, novel_circ_0004892 acted as a ceRNA, modulating MAP3K8 expression via miR-let-7g-5p, thereby influencing innate immunity and steroidogenesis [106].

In aquaculture, Zheng et al. [107] developed a therapeutic circRNA (circRnf103) that alleviated *Vibrio anguillarum* infection and multi-organ damage in fish. This work not only demonstrated the immunoregulatory potential of circRNAs in teleosts, but also proposed circRNA-based immunotherapy as a viable strategy for pathogen control in animal husbandry.

**Figure 5:**
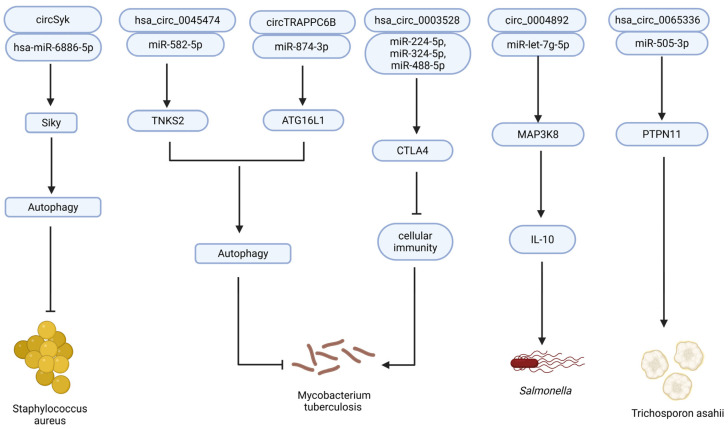
circRNA vaccine in anti-bacterial infection. CircRNAs contribute to antibacterial and antifungal immunity by regulating immune-related signaling pathways. Through miRNA sponging, they modulate key effectors, including Syk, TNKS2, ATG16L1, CTLA4, MAP3K8, and PTPN11, thereby promoting or suppressing autophagy, cytokine production, and cellular immune activity.

Together, these findings underscore the broad utility of circRNAs not only as biomarkers, but also as therapeutic agents across various host–pathogen interactions. Their integration into next-generation vaccine platforms holds promise for more effective and targeted disease prevention strategies (Figure 5).

### 6.3. CircRNA Vaccines Against Fungal Pathogens

CircRNAs are increasingly recognized as key regulators of innate and adaptive immune responses. Certain endogenous circRNAs inhibit protein kinase R (PKR), thereby suppressing innate immune activation and maintaining immune homeostasis [25]. Additionally, circRNAs can promote dendritic cell (DC) differentiation and upregulate immune checkpoint molecules, influencing T cell activation and modulation [108]. These immunomodulatory functions are critical for host defense against fungal pathogens such as *Candida albicans*, *Aspergillus fumigatus*, and *Cryptococcus neoformans*.

Despite the clinical relevance of *Trichosporon asahii* (*T. asahii*), particularly in immunocompromised individuals, where it frequently leads to fatal invasive infections, the molecular basis of its pathogenicity remains poorly understood. RNA-seq analysis has revealed a large number of dysregulated circRNAs following *T. asahii* infection. Notably, Zhang et al. [109] identified hsa_circ_0065336 as a regulatory molecule capable of sponging miR-505-3p, indirectly modulating the expression of PTPN11—a protein known to be essential for proinflammatory cytokine induction [110].

Similarly, in *Aedes aegypti*, fungal infection activates a circRNA–miRNA–mRNA regulatory axis. Novel-circ-930 was shown to modulate the expression of the serine protease ModSP by sponging miRNA-novel-53, thereby affecting host susceptibility to *Beauveria bassiana* infection [111]. These findings underscore the immunomodulatory potential of circRNAs in fungal infections.

CircRNA-based vaccines could be strategically engineered to enhance antifungal immunity by leveraging these endogenous regulatory networks. Such vaccines may modulate critical immune effectors, influence cytokine production, and fine-tune T cell responses through miRNA sponging or gene expression regulation. Given the increasing incidence of fungal infections and the limited efficacy of current antifungal vaccines, circRNA-based immunotherapies represent a promising avenue for innovative vaccine design.

### 6.4. CircRNA Vaccines Against Parasitic Pathogens

Parasitic infections contribute substantially to global morbidity and mortality, often involving intricate host–parasite interactions. Recent studies suggest that circRNAs are involved in the developmental regulation and immune modulation of various parasitic organisms.

In *Caenorhabditis elegans*, circRNAs are expressed throughout different life stages, including gametes, embryos, larvae, and adults, suggesting stage-specific regulatory roles [16,112,113]. In the parasitic nematode *Haemonchus contortus*, more than 20,000 circRNAs have been identified across the infective larvae (L3), adult female (Af), and adult male (Am) stages. Many of these circRNAs possess miRNA-binding sites, highlighting their potential to regulate gene expression through ceRNA mechanisms and modulate host-parasite immune interactions [114].

Similarly, circRNA involvement has been reported in protozoan parasites such as *Trypanosoma brucei* and *Toxoplasma gondii*. In human foreskin fibroblast (HFF) cells infected with *T. gondii*, differential expression of circRNAs was associated with immune signaling pathways, including NOD-like receptor signaling, apoptosis, and NF-κB activation. Among these, circRNA_6:124519352|124575359 was found to modulate miR-146a-5p and miR-150-5p, impacting inflammatory responses and potentially mitigating parasite-induced hepatic damages [115]. Furthermore, circRNAs were found to influence the expression of critical immune mediators such as IL-10 and TREM-1 through their interactions with immune-regulatory miRNAs [116,117].

These discoveries pave the way for the development of circRNA-based vaccines tailored to parasitic infections. By targeting specific host immune pathways, circRNA vaccines could offer more precise immunomodulation compared to traditional protein-based platforms. This precision could reduce off-target effects, minimize disruption to the host microbiota, and provide enhanced protection against diverse parasitic pathogens. As research continues to elucidate the role of circRNAs in parasitic diseases, their application in next-generation vaccine strategies holds significant potential for global health impact.

CircRNA vaccines represent a promising next-generation platform for combating infectious diseases, offering advantages such as improved stability, sustained antigen expression, and low intrinsic immunogenicity. These properties support their application in both therapeutic and prophylactic contexts. Therapeutic constructs have been explored in models of pathogens such as influenza A virus (IAV), hepatitis B virus (HBV), and *Mycobacterium tuberculosis*, targeting chronic or intracellular infections. Prophylactic candidates have focused on pathogens such as SARS-CoV-2, monkeypox virus (MPXV), and Zika virus. These advances collectively suggest that circRNA holds substantial potential to reshape the future of anti-infective vaccine development (Table 1).

## 7. Conclusions and Future Perspectives

CircRNA vaccines represent a promising prophylactic and therapeutic platform against emerging infectious diseases and recrudescent viral pathogens. This innovative modality may further extend to oncotherapeutic applications, potentially revolutionizing both preventive immunization and precision cancer therapy. The burgeoning interest in this field is evidenced by accelerated translational efforts from biopharmaceutical enterprises and academic consortia. However, several critical challenges impede clinical translation. Current circRNA synthesis methodologies are confronted with technical limitations including suboptimal cyclization efficiency and prohibitive reagent costs for key enzymatic components. Moreover, industrial-scale production necessitates standardization and optimization of manufacturing protocols and specialized equipment. Notably, in vitro-transcribed circRNAs demonstrate intrinsic pleiotropic bioactivity that, while potentially advantageous for immune activation, carries risks of off-target interactions and unanticipated immunogenic sequelae. This biological complexity mandates stringent sequence engineering guided by computational modeling, coupled with multidimensional assessment of toxicological profiles and immunogenic characteristics. Persistent technical barriers in scalable production systems, tunable immunomodulation strategies, and stringent quality control protocols collectively constrain circRNA vaccine development to preclinical validation stages, particularly regarding anti-infectious disease applications. Moving forward, interdisciplinary integration of nucleic acid nanotechnology, immunoinformatics, and advanced delivery systems will be imperative to realize the full clinical potential of this novel vaccine paradigm.

Optimization strategies targeting enhanced protein expression, diminished immunogenicity, and augmented molecular stability constitute pivotal objectives in circRNA vaccine development. The internal ribosome entry site (IRES) operates as the principal translational regulatory element in circRNA architectures, exerting a profound influence on protein expression levels. Initial breakthroughs came from Wesselhoeft et al. [27], who characterized the CVB3 IRES as exhibiting superior translational competence in 2018. Subsequent investigations revealed that HRV-B3 IRES surpassing previous variants in efficiency through systematic screening [19]. Most recently, Yu et al. [118] established EV-A IRES as the optimal performer across multiple cellular models, including DC2.4, THP-1, and HEK293T cells. Concurrently, artificial intelligence has emerged as a transformative tool for IRES functionality prediction, as exemplified by Zhou et al.’s [119] development of DeepCIP—a deep learning framework demonstrating superior accuracy in circRNA IRES profiling compared to conventional algorithms.

Notably, while N1-methyl-pseudouridine (m1Ψ) confers enhanced translational capacity and attenuated immune activation in linear mRNA vaccines, its implementation in circular RNA systems paradoxically compromises functionality [18]. In contrast, endogenous circRNA studies have illuminated N6-methyladenosine (m6A) modification as a potent translation enhancer through recruitment of eukaryotic initiation factors. Building upon these findings, Chen et al. [19] engineered circRNAs with 5% m6A incorporation, achieving comparable protein yields to native constructs while significantly reducing immunogenic potential. However, m6A modification may simultaneously predispose circRNAs to endoribonucleolytic cleavage within cytoplasmic compartments, implying a dual role in modulating in vivo stability. This mechanistic duality necessitates rigorous exploration through integrated structural biology and pharmacokinetic studies.

A persistent technical hurdle stems from the inherent structural instability of circRNA molecules. Despite their widespread application, conventional purification methodologies including high-performance liquid chromatography (HPLC) and gel electrophoresis demonstrate limited capacity to resolve nicked or fragmented RNA species. Emerging evidence [18] elucidates that both intact circRNA and process-related impurities engage with pattern recognition receptors (PRRs), thereby triggering unintended immune activation. Mechanistically, cellular surveillance systems discriminate exogenous RNA through terminal structure recognition and double-stranded RNA (dsRNA) detection mechanisms. Notably, intramolecular folding patterns within IRES domains—particularly complex secondary structures—may serve as cryptic pathogen-associated molecular patterns, potentiating innate immune signaling. This immunological paradox presents context-dependent implications: whereas uncontrolled immunostimulation poses clinical risks for prophylactic vaccines against infectious pathogens, therapeutic cancer vaccines strategically leverage these adjuvant-like properties to amplify antitumor immunity [120]. For example, recent studies have shown that small circRNA vaccines encoding tumor-associated antigens can induce durable and potent CD8^+^ and CD4^+^ T cell responses in murine melanoma models, particularly when combined with immune checkpoint inhibitors [121].

In addition to its impact on innate immune sensing, the secondary and tertiary folding of circRNA significantly modulates translational accessibility, RNA stability, and antigen expression kinetics. For example, optimized folding that exposes internal ribosome entry sites (IRESs) and start codons can enhance cap-independent translation, while overly compact configurations may sterically occlude essential translational elements [118]. Qu et al. [5] demonstrated that a SARS-CoV-2 circRNA vaccine encoding the RBD achieved significantly improved translation efficiency and neutralizing antibody titers when destabilizing mutations were introduced near the IRES and AUG start codon to reduce local structural rigidity. Moreover, well-defined stem-loop structures can shield circRNAs from exonucleolytic degradation, thereby prolonging their intracellular half-life, an essential feature for sustained antigen presentation in vaccine contexts. However, these structural benefits must be carefully balanced, as excessive double-stranded regions can activate dsRNA sensors such as PKR, leading to translational repression or proinflammatory signaling [25].

Therefore, the rational design of circRNA vaccines must incorporate structure-informed strategies that modulate folding to achieve optimal immunogenicity, stability, and translation. This entails a multifaceted approach encompassing sequence engineering, structural modeling, site-specific chemical modifications, and rigorous purification protocols. Achieving this balance is critical for the clinical translation of circRNA vaccines, particularly in infectious disease settings where both efficacy and safety are paramount.

Optimization of circularization methodologies constitutes a pivotal focus for advancing circRNA production efficiency. A critical limitation emerges, as circularization efficiency exhibits an inverse correlation with precursor RNA length, imposing critical constraints on vaccine construct design. Among existing approaches, permuted intron–exon (PIE) systems utilizing group I introns remain predominant due to their robust ligation capacity for extended RNA sequences. However, this strategy invariably introduces exogenous sequences, predisposing the resultant circRNA to spurious RNA duplex formation that may aberrantly activate innate immunity and compromise structural fidelity. Alternative techniques including T4 RNA ligase-mediated and chemical ligation protocols produce pharmaceutical-grade circRNA with minimal byproducts, yet suffer from deleterious length-dependent attenuation in ligation efficiency. Notably, group II intron-based circularization has emerged as a transformative strategy, enabling generation of topologically pristine circRNA devoid of extraneous nucleotides. Furthermore, recircularization technology—where primary ligation products undergo secondary cyclization—demonstrates potential to amplify production yields while facilitating industrial-scale manufacturing [27]. Methodological breakthroughs in these domains will critically determine three cardinal parameters of vaccine performance: enhanced structural integrity, prolonged antigen expression kinetics, and optimized immunogenic potency against target pathogens.

Refinement of purification protocols constitutes a critical determinant in acquiring pharmaceutical-grade circRNA. Residual synthetic byproducts persisting through suboptimal purification exhibit potent immunostimulatory properties, predisposing vaccines to off-target inflammatory sequelae and accelerated RNA catabolism. These contaminants not only jeopardize biosafety profiles, but also dysregulate translational fidelity, ultimately undermining therapeutic efficacy. Therefore, achieving ultra-pure circRNA preparations is paramount to minimizing aberrant immune activation, extending pharmacokinetic stability, and maximizing clinical performance. Recent evidence underscores the heterogeneous and immunogenic nature of these synthetic impurities, including double-stranded RNA (dsRNA), linear RNA precursors, nicked RNA species, and fragmented RNA molecules, demonstrating robust activation of innate immune sensors such as Toll-like receptors (TLR3, TLR7, TLR8), RNA-dependent protein kinase R (PKR), OAS, MDA5, and RIG-I, thereby triggering downstream inflammatory cascades involving NF-κB, TNF signaling, and cytokine release. Notably, even trace amounts of these contaminants are sufficient to provoke significant immune activation, highlighting the critical importance of achieving ultra-pure circRNA preparations to minimize aberrant immune responses, extend pharmacokinetic stability, and maximize clinical performance. Current purification methodologies—spanning gel electrophoresis, enzymatic digestion (RNase R/RNase H), and high-performance liquid chromatography (HPLC)—each present unique constraints. Notably, HPLC demonstrates superior high-resolution separation capacity and manufacturing scalability, yet exhibits intrinsic limitations in discriminating linear RNA isoforms with analogous molecular weights [18]. Moreover, the sole application of RNase R fails to achieve comprehensive elimination of residual linear species, necessitating combinatorial enzymatic processing. For instance, preliminary phosphatase treatment prior to RNase R digestion synergistically reduces immunogenic potential, albeit at the cost of escalated procedural complexity that impedes industrial-scale implementation. To address these limitations, recent studies have validated advanced purification strategies, such as multi-step protocols combining enzymatic treatments with cellulose-based chromatography. These methods more effectively remove dsRNA and other immunogenic impurities, thereby reducing innate immune activation and improving circRNA purity, stability, and yield. However, their large-scale implementation still requires further optimization in procedural simplicity, reproducibility, and cost efficiency [122]. Future progress mandates establishment of internationally harmonized reference standards for dsRNA contaminants, incorporating sequence-specific physicochemical parameters such as nucleobase composition, polymer length, 5′-terminal phosphorylation states, and epigenetic modification patterns. Implementation of such standardized frameworks will prove indispensable for maintaining batch-to-batch consistency, fulfilling regulatory requirements, and accelerating clinical translation in circRNA vaccine development [123].

Finally, improving the safety profile of circRNA vaccines remains a central concern. While endogenous circRNAs play essential roles in gene regulation, transcription, and cell signaling—and some even encode functional proteins—the immunogenicity of engineered circRNAs varies depending on production methods. For instance, group I intron-based circularization systems may leave residual intron sequences, known as “scars”, that elicit immune responses [32]. Chen et al. [26] demonstrated that circRNAs constructed using the PIE method with the T4 td gene intron activated multiple immune-related genes, whereas circRNAs using the ZKSCAN1 intron did not, suggesting that the choice of intron sequences significantly affects immunogenicity. Beyond intronic scars, dsRNA contamination is another potential concern. For example, dsRNA residues have been implicated in mRNA vaccine-associated myocarditis, raising questions about whether similar risks exist with circRNA vaccines [124]. Given that circRNA synthesis may also produce dsRNA byproducts, further research is necessary to evaluate their safety implications. Additionally, unintended translation products derived from cryptic open reading frames or noncanonical start sites within circRNAs represent an emerging safety consideration. Although such off-target peptides have not been widely reported, they may theoretically trigger unforeseen immune responses or interfere with antigen specificity [125]. To address this, circRNA vaccine development pipelines should incorporate rigorous profiling techniques such as ribosome footprinting, proteomic mass spectrometry, and luciferase-based reporter assays to verify translational fidelity and eliminate aberrant expression. Moving forward, reducing intron retention, eliminating dsRNA contaminants, and refining purification methods will be crucial to enhance the safety and clinical viability of circRNA vaccines.

## Figures and Tables

**Table 1:** circRNA vaccines in anti-pathogen infection.

Potential Vaccine Type	Pathogen Category	Pathogen	Encoded Antigen or Molecular Target	Development Stage	Reference
Therapeutic	Viruses	SARS-CoV-2	hsa_circ_0000479	in vitro + in silico	[59]
Therapeutic/Prophylactic			RBD antigens	in vivo + in vitro	[5]
Therapeutic		Influenza A virus (IAV)	circ-GATAD2A	in vitro	[69]
Therapeutic			circRNA_0050463	in vitro	[70]
Therapeutic			AIVR	in vitro	[71]
Therapeutic			CircMerTK	in vitro	[72]
Therapeutic			circVAMP3	in vivo + in vitro	[73]
Prophylactic		Zika virus	EDIII-Fc, NS1	in vivo + in vitro	[77]
Therapeutic		Hepatitis B virus	hsa_circ_0005389	in silico + in vitro	[79]
Therapeutic			hsa_circ_0000038	in vitro	[80,81]
Therapeutic			circ_00004812	in vitro + vivo	[82]
Therapeutic		Hepatitis C virus	Artificial circular RNA sponge targeting miR-122	in vitro	[83]
Therapeutic		*B. mori* cypovirus (BmCPV)	vcircRNA_000048	in vitro	[86]
Therapeutic/Prophylactic		Monkeypox virus (MPXV)	cirA29L, cirA35R, cirB6R, cirM1R.	Phase I (ongoing)	[87]
Therapeutic	Bacteria	*Staphylococcus aureus*	hsa_circ_0002483	in vitro	[93]
Therapeutic		*Staphylococcus aureus*	circSyk	in vivo + in vitro	[94]
Therapeutic		*Mycobacterium tuberculosis*	hsa_circ_0045474	in vivo + in vitro	[96]
Therapeutic			circTRAPPC6B	in vitro	[97]
Therapeutic			hsa_circ_0003528	in vivo + in vitro	[98]
Therapeutic			circAGFG1	in vivo + in vitro	[100]
Therapeutic			Hsa_circ_0007919	in vitro	[101]
Therapeutic		*Escherichia coli*	Hsa_circ_0001021	in vivo + in vitro	[105]
Therapeutic		*Salmonella enteritidis*	circ_0004892	in vivo + in vitro	[106]
Therapeutic		*Vibrio anguillarum*	circRnf103	in vivo	[107]
Therapeutic	Fungi	*T. asahii*	hsa_circ_0065336	in vitro	[109]
Therapeutic		*B. bassiana*	novel-circ-930	in vivo	[111]
Therapeutic	Parasites	*Toxoplasma gondii*	circRNA 6:124519352|124575359	in vivo	[115]

## Supplementary Materials

The following supporting information can be downloaded at: https://www.mdpi.com/article/10.3390/vaccines13060563/s1, Table S1: Distinct Advantages and Disadvantages of circRNA Vaccines Compared to Other Vaccine Platforms; Table S2: Comparative Overview of circRNA Circularization Techniques. References [2,19,23,31,32,33,34,35,126,127,128,129,130,131,132,133,134,135,136,137] are cited in the Supplementary Materials.
