# Supplementary material for: Rational Design and Immunological Mechanisms of Circular RNA-Based Vaccines: Emerging Frontiers in Combating Pathogen Infection
Source: Vaccines (Basel). 2025 May 26;13(6):563. doi: 10.3390/vaccines13060563 (PMC12197763; doi:10.3390/vaccines13060563)
Supplement: Supplementary file 1 [file vaccines-13-00563-s001.zip › vaccines-3618101-supplementary.pdf]

Table S1. Distinct Advantages and Disadvantages of circRNA Vaccines Compared to Other Vaccine Platforms

Vaccine Platform	Advantages	Disadvantages	Reference
circRNA	Low immunogenicity；<br>Potentially longer-lasting immunity；<br>Higher stability compared to linear RNA；<br>Rapid, scalable manufacturing；	Novel platform, limited clinical data；<br>Regulatory uncertainties；<br>Delivery optimization challenges；	[2, 19]
mRNA	Rapid development and production；<br>High immunogenicity and efficacy；<br>Flexible and adaptable design；	Requires ultra-cold storage (-70°C)；<br>Poor stability,easily degradable<br>Potential reactogenicity (side effects)；	[126, 127]
DNA Vaccine	Can be stored at room temperature<br>Simple production process low cost,can mass production	Requires nuclear entry for transcription, limiting efficiency;<br>Potential risk of genomic integration ;<br>Theoretical concern of insertional mutagenesis or oncogenesis;	[128, 129]
Inactivated Vaccine	Excellent safety profile；<br>Stable at refrigerated temperatures；<br>Established manufacturing methods；	Lower immunogenicity (often requires adjuvants and boosters)；<br>Longer production time；	[130, 131]
Live-attenuated Vaccine	Strong, long-lasting immunity；<br>Usually single-dose efficacy；<br>Stimulates robust cellular and humoral immunity；	Risk of reversion to pathogenic form；<br>Not suitable for immunocompromised；<br>individuals<br>Cold-chain storage requirements；	[132, 133]
Subunit Vaccine	Excellent safety, no risk of infection；<br>Highly stable and easy storage；<br>Suitable for immunocompromised people；	Moderate to low immunogenicity (requires strong adjuvants)；<br>Generally requires multiple doses for effective immunity；	[134, 135]
Toxoid Vaccine	Well-established safety profile；	Only protects against toxin-producing	[136]

	Good stability, easy storage and transport；<br>Reliable immune response to specific toxin-mediated diseases；	pathogens；<br>Requires regular boosters；<br>Does not protect against pathogen infection itself；	
Viral Vector Vaccine	High immunogenicity, including robust cellular response；<br>Induce lasting immunity；<br>Flexible antigen encoding capacity；	Pre-existing immunity to viral vectors can reduce efficacy；<br>Potential safety concerns (rare adverse events)；<br>Complex manufacturing；	[137]

Table S2. Comparative Overview of circRNA Circularization Techniques.

Characteristics	Chemical Synthesis	Enzymatic Synthesis	Ribozyme-Mediated Circularization
Principle	Uses chemical linkers to join functionalized RNA ends	Uses RNA/DNA ligases to covalently link termini	Utilizes self-splicing ribozyme elements to mediate ligation
Materials	Modified nucleotides, chemical catalysts	Ligases, ATP, splint	Self-splicing introns
Linkage Type	Phosphodiester or non-natural bonds	Enzymes catalyze nucleotidyl transfer reactions to form phosphodiester bonds	Phosphodiester bonds via two-step transesterification
Efficiency	Low	High for moderate-length RNAs	High for moderate-length RNAs
Yield & Purity	Requires HPLC for purification; side products include non-circular or misfolded RNA	High with optimized splinting; free of extraneous sequences	High; PIE-based systems yield scarless circRNAs with error rates <0.01%

Scalability	Low; high cost and low yield	Moderate; suitable for small- to medium-scale production	High; industrial-scale production demonstrated using Clean-PIE and ECRR platforms
Safety	Uses toxic or hazardous reagents (e.g., BrCN); 2′,5′ linkages may affect folding and function	Generally safe; no toxic reagents used; immunogenically silent output	Minimal biosafety concerns; scarless design avoids innate immune activation
Optimization Needs	Requires careful design of linkers and reaction conditions for high efficiency and purity	Requires RNA end pre-orientation; reaction condition optimization is critical	Requires ribozyme sequence context design; careful optimization needed to avoid scar sequences
Limitations	Low throughput, unsuitable for >70 nt; noncanonical linkages distort RNA structure	Side reactions (oligomerization); reduced efficiency with long or structured RNA; splint design adds complexity	May leave scar or residual sequence in suboptimal systems; complex design logic for sequence context and secondary structure
Applications	Short circRNAs (<70 nt) for in vitro studies or chemical probes	Vaccine-grade circRNAs up to ~500 nt; low innate immunogenicity desirable for therapeutic use	Benchmark platform for high-purity, scalable circRNA vaccines; gold standard in industrial applications (e.g., >mg-scale Clean-PIE manufacturing)
Reference	[23]	[31, 32]	[33-35]
